# Perceiving like a Bat: Hierarchical 3D Geometric–Semantic Scene Understanding Inspired by a Biomimetic Mechanism

**DOI:** 10.3390/biomimetics8050436

**Published:** 2023-09-19

**Authors:** Chi Zhang, Zhong Yang, Bayang Xue, Haoze Zhuo, Luwei Liao, Xin Yang, Zekun Zhu

**Affiliations:** College of Automation Engineering, Nanjing University of Aeronautics and Astronautics, Nanjing 211106, China; laozhang@nuaa.edu.cn (C.Z.);

**Keywords:** biomimetic, SLAM, scene understanding, 3D reconstruction, attention mechanism, semantic navigation

## Abstract

Geometric–semantic scene understanding is a spatial intelligence capability that is essential for robots to perceive and navigate the world. However, understanding a natural scene remains challenging for robots because of restricted sensors and time-varying situations. In contrast, humans and animals are able to form a complex neuromorphic concept of the scene they move in. This neuromorphic concept captures geometric and semantic aspects of the scenario and reconstructs the scene at multiple levels of abstraction. This article seeks to reduce the gap between robot and animal perception by proposing an ingenious scene-understanding approach that seamlessly captures geometric and semantic aspects in an unexplored environment. We proposed two types of biologically inspired environment perception methods, i.e., a set of elaborate biomimetic sensors and a brain-inspired parsing algorithm related to scene understanding, that enable robots to perceive their surroundings like bats. Our evaluations show that the proposed scene-understanding system achieves competitive performance in image semantic segmentation and volumetric–semantic scene reconstruction. Moreover, to verify the practicability of our proposed scene-understanding method, we also conducted real-world geometric–semantic scene reconstruction in an indoor environment with our self-developed drone.

## 1. Introduction

Scene understanding has gained increasing attention in the biomimetic and robotics community as a means to help intelligent robots perceive the world. High-level environmental perception is a precondition for robot autonomous navigation in an unexplored environment and for efficient human–computer interaction. The next generation of agents must be able to understand and fulfill high-level commands. For example, users can directly give high-level instructions to the agent through dictation: “Come here and hand over this article to Professor Lee at the engineering practice center”. Agents must holistically understand the scene and quickly draw up the optimal execution plan.

In recent years, some studies have proposed partial solutions to these problems, such as simultaneous localization and mapping (SLAM) [1,2,3,4,5,6], autonomous path replanning [7,8,9,10], pattern recognition [11,12,13,14], and iterative reconstruction [15,16,17]. Nevertheless, research in these fields has traditionally proceeded in isolation, and there is currently no complete full-stack solution for scene parsing. Scene understanding in unknown environments still remains an enormous challenge because of the time-varying situations and stringent requirements for computing power, system latency, and energy consumption caused by the limited resources of agents.

**Figure 1:**
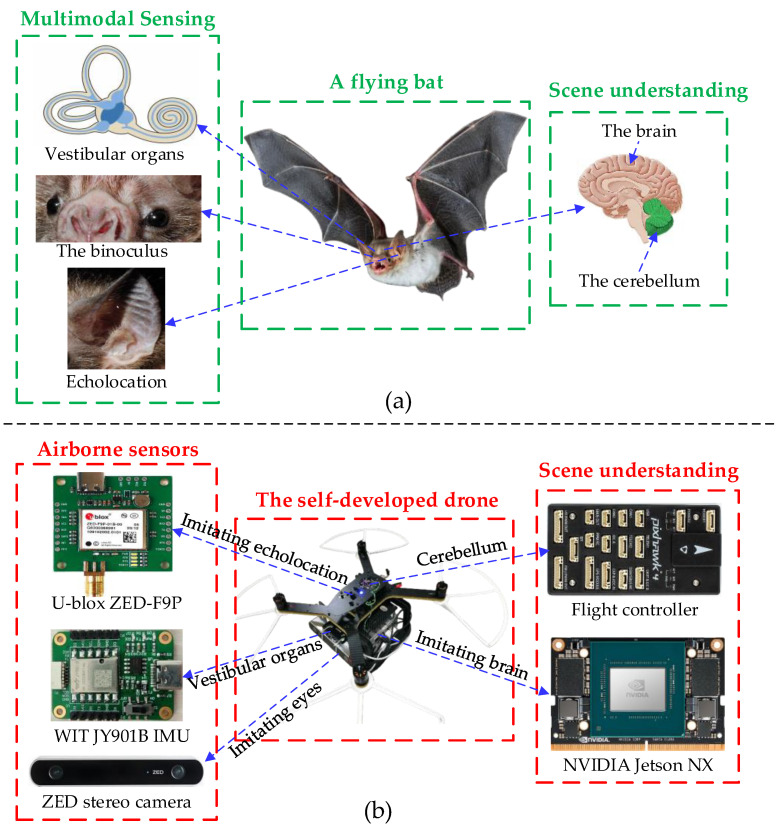
The comparison of the scene-understanding mechanism between bats and robots. (**a**) Bats can perceive the surrounding environment with their vestibular organs, visual perception, echolocation, and spatiotemporal analysis systems. (**b**) Robots can perceive the environment with a set of elaborate biomimetic sensors and a brain-inspired parsing algorithm related to scene understanding.

To surmount the above challenges, we turned to nature for inspiration. Humans and animals have shown us their incredible environment perception capabilities and autonomous navigating abilities in a large-scale complex environment [18,19,20,21,22]. As humans, we understand the surrounding environments effortlessly: we receive and transmit high-level instructions and draw up long-distance travel between different cities, and even accurately predict what will happen in the future. When animals are sensing the environment and generating navigation maps, different sensory cues can activate multiple types of sensory cells in their head [23,24,25], as illustrated in Figure 1. Animal neurons can quickly structure spatiotemporal relationships for the surrounding environment [26,27,28,29,30,31]. This is in stark contrast with today’s machine capabilities; machines only receive navigation instructions with Cartesian coordinate systems, and do not have inference algorithms to achieve high-level decision making at multiple levels of abstraction.

This article presents a neuromorphic general scene-understanding system that emulates the state estimation system, visual perception system, spatiotemporal analysis system, and echolocation system of a bat to efficiently and comprehensively perceive the environment. Although developing and applying a robot scene-understanding system that completely includes all these ingredients can only be the purpose of a long-term research agenda, we attempt to provide the first step towards this aspiration by proposing a biologically inspired environmental perception strategy, and validating it through extensive experiments. The main novelties of the proposed scene-understanding system are exhibited below:

- Inspired by bat’s binoculus and vestibular organs, we present a lightweight and drift-free visual–inertia–GNSS tightly coupled multisensor fusion (LDMF) strategy for unmanned aerial vehicle (UAV) pose estimation, which achieves accurate pose estimation by imitating the state estimation system of bats.
- Inspired by the bat’s optic nervous system, we innovatively designed a neural network sub-module with a neuro-like attention mechanism. Based on this, we constructed a novel Transformer–CNN hybrid neural architecture, dubbed BatNet, for real-time pixel-level semantic segmentation.
- Inspired by the bat’s spatiotemporal analysis and echolocation system, we propose a layered directed scene graph called a hierarchical scene graph (HSG) to represent the spatiotemporal relationships between substances, and implement a truncated signed distance field (TSDF) to obtain the volumetric scene mesh at each keyframe.

## 2. System Overview

**Figure 2:**
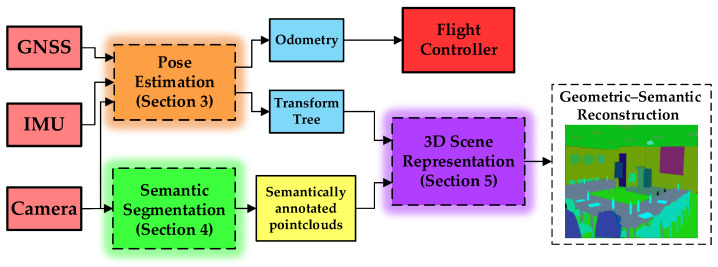
The flowsheet of the proposed bat-inspired scene-understanding system.

As diagrammed in Figure 2, we use the ZED binocular camera, WIT JY901B inertial measurement unit, and ZED-F9P GNSS receiver to simulate the bat perception organs such as the binocular organs, microscopic canals, saccules, and ears. Additionally, the hybrid neural networks, HSG, and TSDF are used to replace the visual nerve center and spatiotemporal analysis system in the bat brain. The ZED stereo camera can directly output color images and corresponding depth images. The different types of sensor information are tightly coupled (expounded in Section 3) to provide a global consistent UAV odometry and coordinate transformation tree. The three-dimensional volumetric scene reconstruction leverages a TSDF-based strategy to generate the global scene mesh. At each keyframe, we capture depth images from the ZED stereo camera and convert color and depth images into spatial pointcloud data. Then, we perform the truncated signed distance field (proposed in Section 5.2) to obtain the volumetric scene mesh at each keyframe.

For geometric–semantic scene reconstruction, we make use of a pixel-level image semantic segmentation method, BatNet (proposed in Section 4), to categorize each image pixel, then semantically annotate the global scene mesh. Furthermore, we exploit a hierarchical scene graph (proposed in Section 5.1) to represent the spatiotemporal relationships between substances. During the packaged projection, we also semantically propagate a label to each spatial pointcloud generated by the ZED stereo camera. After packaged semantic projection, each spatial voxel has a vector of category probabilities, which is completely consistent with the category in HSG.

## 3. Bat-Inspired State Estimation

**Figure 3:**
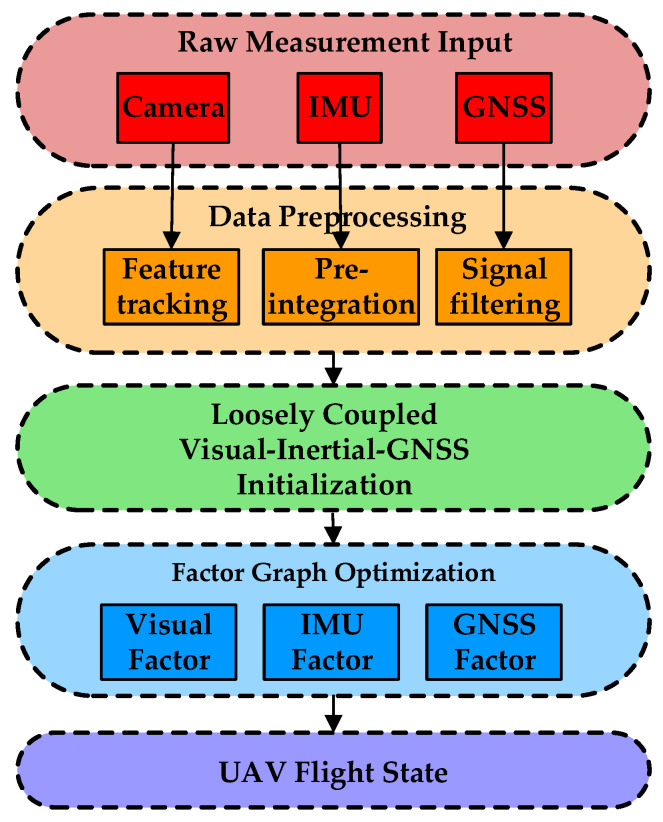
The flowsheet of the LDMF state estimation system.

Inspired by the bat’s pose estimation system, we present a lightweight and drift-free vision–IMU–GNSS tightly coupled multisensor fusion (LDMF) strategy for UAV state estimation, as shown in Figure 3. The multisensor fusion is formulated as a probabilistic factor graph optimization, and the whole system states inside the circumscribed container can be summarized as follows:

(1) $$\left\{\begin{array}{l} \chi ={\left[x_{0},x_{1},\;\ldots ,\;x_{n},\lambda_{1},\lambda_{2},\ldots ,\lambda_{m},\psi \right]}^{T}\\ x_{k}={\left[o_{r_{tk}}^{w},v_{r_{tk}}^{w},p_{r_{tk}}^{w},b_{\omega_{tk}},b_{a_{tk}},\delta t,\delta t^{\prime }\right]}^{T},\;k\in \left[0,n\right] \end{array}\right.$$

where *x_k_* is the robot state at the time *t_k_* that the *k*th image frame is captured. It contains nose orientation $o_{r_{tk}}^{w}$, velocity $v_{r_{tk}}^{w}$, position $p_{r_{tk}}^{w}$, gyroscope bias $b_{\omega_{tk}}$, and acceleration bias $b_{a_{tk}}$. δt and $\delta t^{\prime }$ correspond to the clock biases and bias drifting rate of the GNSS receiver, respectively. *n* is the sliding window size and *m* is the total number of visual features in the sliding window. $\lambda_{l}$ is the inverse depth of the *l*th visual feature. ψ is the yaw bias between the odometry and the world frame.

Assuming that the measurement noise conforms to a Gaussian distribution with zero mean, then the solution process of the UAV state vector χ can be expressed as:

(2) $$\begin{array}{cc} \chi & =\underset{\chi }{\arg\;\max}\;P\left(\chi |z\right)\\ & =\underset{\chi }{\arg\;\min}\left({\left\|e_{p}-H_{p}\chi \right\|}^{2}+\sum_{k=1}^{n}{\left\|E\left(z_{k},\chi \right)\right\|}^{2}\right) \end{array}$$

where *z* is the UAV pose linear observation model, *H_P_* matrix means the prior UAV pose information obtained by the airborne camera, *n* is the number of UAV state vectors, and *E*(·) implies the sum of all sensor measurement error factors.

Finally, the UAV pose can be obtained by optimizing the UAV state vector χ by employing probability factor graph optimization. For the detailed process of factor graph optimization, please refer to our previous literature [32,33,34,35].

## 4. Bat-Inspired Real-Time Semantic Segmentation

In this section, we first revisit the original self-attention and external-attention mechanisms, and provide detailed elaboration of our ingenious bionics-inspired attention mechanism. Then we drive two bionics-inspired attention modules to compose an artificial neural block. Finally, a complete neural network dubbed BatNet is constructed for real-time semantic segmentation.

### 4.1. Attention Mechanism

#### 4.1.1. Self-Attention Mechanism

**Figure 4:**
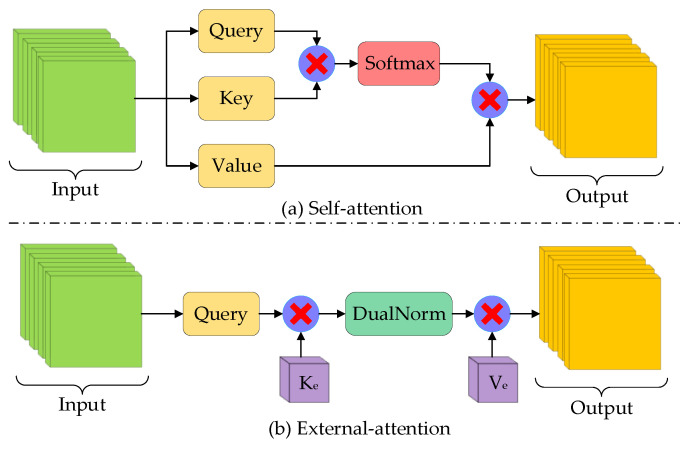
Comparison between self-attention and external-attention.

In neuroscience, due to bottlenecks in information processing, bats selectively focus on a portion of all information while ignoring other unimportant parts. In situations where computing resources are limited, attention mechanisms can automatically allocate computing resources to more important tasks. A self-attention (SA) module can be considered as representing an input query and a set of key–value pairs from the input itself to an output, where both input and output are vectors. Given an input feature matrix $M\in {\mathbb{R}}^{N\times d_{}}$, where *N* is the number of pixels in the feature matrix and *d* represents the feature map dimensions, self-attention simultaneously projects the input feature to a query matrix $Q\in {\mathbb{R}}^{N\times dq}$, a key matrix $K\in {\mathbb{R}}^{N\times d_{k}}$, and a value matrix $V\in {\mathbb{R}}^{N\times d_{v}}$, as shown in Figure 4a. The matrix of attention outputs can be formulated as:

(3) $$\mathrm{SA}\left(Q,K,V\right)=\mathrm{Softmax}\left(\frac{QK^{T}}{\sqrt{dq_{}}}\right)V$$

The value of *d_q_* is usually relatively large, thus bringing the SoftMax function into regions where it has extremely small gradients. To alleviate this negative impact, the inner products is divided by $\sqrt{dq}$.

**Figure 5:**
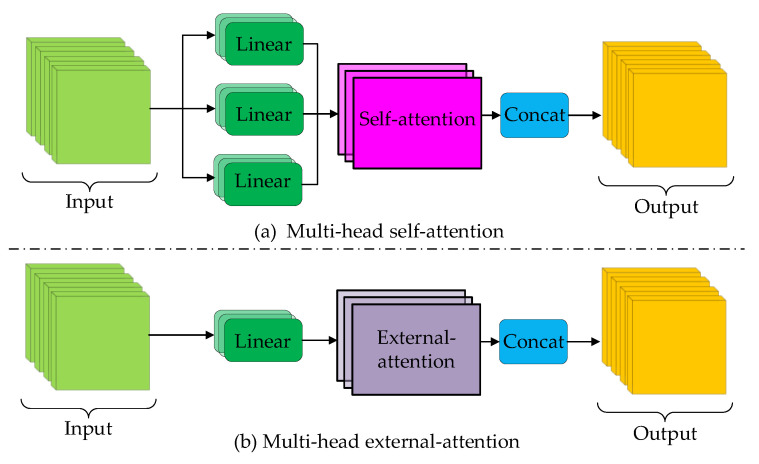
Comparison between multi-head self-attention and multi-head external attention.

Instead of performing a single matrix multiplication operation, it is better to divide the input into several equal parts and respectively project the *Q*, *K*, and *V* matrices *H* times with a learnable weighting matrix. Their respective attention maps are then calculated in parallel, which is named multi-head self-attention (MHSA), as illustrated in Figure 5a. The multi-head self-attention structure allows the neural network to aggregate semantic information from different representation subspaces at different positions. In practice, the multi-head mechanism is similar to partitioned matrix multiplication. Multi-head self-attention can capture different affinities between input vectors, promoting the multimodal performance of neural networks to a certain extent. The computational process of multi-head self-attention can be formulated as:

(4) $$\left\{\begin{array}{c} \mathrm{MHSA}\left(Q,K,V\right)=\mathrm{Concat}\left({\mathrm{head}}_{1},\;\ldots {,\;\mathrm{head}}_{H}\right)W^{O}\\ {\mathrm{head}}_{i}=\mathrm{SA}\left(QW_{i}^{Q},KW_{i}^{K},VW_{i}^{V}\right) \end{array}\right.$$

where the symbol head*_H_* represents the input channel, *H* is the number of heads, and *i* is the *i*th head. The symbols $W_{i}^{Q}$, $W_{i}^{K}$, $W_{i}^{V}$, and $W^{O}$ are the shared parameter matrices.

#### 4.1.2. External-Attention Mechanism

Although the multi-head mechanism can parallelize the matrix multiplication operation to some extent, the quadratic complexity still remains. Furthermore, the self-attention mechanism only utilizes the relative relationship between an input batch, while ignoring the potential correlations in the entire training dataset, which implicitly limits the model flexibility and generality.

To address these disadvantages, a flexible and lightweight attention mechanism called external attention (EA) was invented, which manufactures an attention map between a query matrix *Q_e_* and two different learnable memory units, *K_e_* and *V_e_*, as the key and value, respectively. The illustration of external attention is shown in Figure 4b. The memory unit is an external parameter matrix independent of the query matrix, which acts as a prior and traverses the whole sample. The external-attention mechanism can be expressed by the following formula:

(5) $$\mathrm{EA}\left(Q_{e},K_{e},V_{e}\right)=\mathrm{DualNorm}\left(Q_{e}K_{e}{}^{T}\right)V_{e}$$

where $Q_{e}\in {\mathbb{R}}^{N\times d}$ is the input query, $K_{e},V_{e}\in {\mathbb{R}}^{S\times d}$ are the shared external learnable parameter matrices, and *S* is the dimension of the parameter matrix. The symbol DualNorm represents the double normalization operation proposed by Yu et al. [36], which normalizes the columns and rows in an attention map separately.

Figure 5b shows the multi-head version of the external-attention mechanism. The multi-head external attention (MHEA) mechanism can be written as:

(6) $$\left\{\begin{array}{c} \mathrm{MHEA}\left(Q_{e},K_{e},V_{e}\right)=\mathrm{Concat}\left({\mathrm{head}}_{1},\;\ldots {,\;\mathrm{head}}_{H}\right)W^{O}\\ {\mathrm{head}}_{i}=\mathrm{EA}\left(Q_{e}{}^{i},K_{e}^{\prime },V_{e}^{\prime }\right) \end{array}\right.$$

where, as in Formula (4), the symbol head*_i_* represents the input channel, *H* is the number of heads, and *i* is the *i*th head. $K_{e}^{\prime },V_{e}^{\prime }\in {\mathbb{R}}^{S\times d^{\prime }}$ are the shared multi-head parameter matrices, $d^{\prime }=d/H$. Although the external-attention mechanism leverages shared parameter matrices to calculate the attention map corresponding to different heads, the system latency caused by excessive matrix computation still remains.

#### 4.1.3. Neuro-like Attention Mechanism

**Figure 6:**
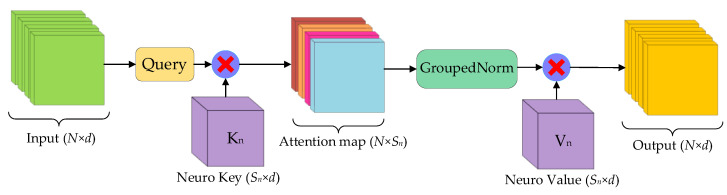
The neuro-like attention structural diagram.

To mitigate the impact of multi-head attention, we drew inspiration from neuroscience and redesigned a concise but efficient attention mechanism called neuro-like attention (NLA), as shown in Figure 6. NLA inherits the linear complexity from the EA mechanism, and dispenses with the multi-head mechanism to reduce the system latency caused by restricted video RAM bandwidth on neural computing platforms. The neuro-like attention mechanism can be expressed as:

(7) $$\mathrm{NLA}\left(Q_{n},K_{n},V_{n}\right)=\mathrm{GroupedNorm}\left(Q_{n}K_{n}{}^{T}\right)V_{n}$$

where $Q_{n}\in {\mathbb{R}}^{N\times d}$ is the neuro-like attention input query matrix and $K_{n},V_{n}\in {\mathbb{R}}^{S_{n}\times d}$ are the external learnable parameter matrices, $S_{n}=S\times H$. GroupedNorm denotes the grouped normalization operation, which distributes the original double normalization into *H* channels.

It is worth noting that for image processing, the hyperparameter *S* is usually much smaller than *N* (typically *N* = 512 × 512 = 262,144, while *S* = 64). Thus, neuro-like attention has lower computational complexity compared to self-attention, allowing it to be directly deployed on mobile devices. Compared with external attention, the neuro-like attention mechanism has several inherent advantages. Firstly, neuro-like attention dispenses with the multi-head structure from the EA mechanism. Therefore, the system output latency caused by restricted video memory bandwidth is reduced. As an alternative, neuro-like attention utilizes grouped normalization operations to maintain the superiority of MHEA to a certain extent. Secondly, the neuro-like attention mechanism expands the number of learnable parameters in external memory units by a quadratic factor of *H*. Thus, having more parameters provides more substantial analytical capabilities for scene-understanding tasks. Finally, the neuro-like attention mechanism integrates the sequential matrix manipulation, which significantly reduces the number of linear matrix operations and is quite suitable for the neuro-like device architecture.

#### 4.1.4. Dual-Resolution Attention Mechanism

The feature map fusion structure with different resolutions has achieved incredible effects in image semantic segmentation tasks. Multi-resolution feature fusion includes two branches: high-resolution thread and low-resolution thread. The high-resolution branch excels in extracting detailed information such as geometric textures from input images. Since the low-resolution branch has a larger receptive field than its counterpart, it focuses on aggregating global semantic information. In order to incorporate global contextual information from the low-resolution branch into the high-resolution branch, we heuristically designed an imaginative attention mechanism, dubbed dual-resolution attention (DRA). The calculation can be represented by the following formula:

(8) $$\left\{\begin{array}{c} \mathrm{DRA}\left(Q_{d},K_{d},V_{d}\right)=\mathrm{Softmax}\left(\frac{Q_{d}K_{d}{}^{T}}{\sqrt{d_{q}}}\right)V_{d}\\ K_{d},V_{d}=\psi \left(Q_{n}\right) \end{array}\right.$$

where $Q_{d}$ is the DRA module input query, $K_{d},V_{d}\in {\mathbb{R}}^{S\times d}$ are the external parameter matrices, *d_q_* means the feature dimension of $Q_{d}$, and the symbol ψ implies convolution, pooling, and permutation matrix manipulations.

**Figure 7:**
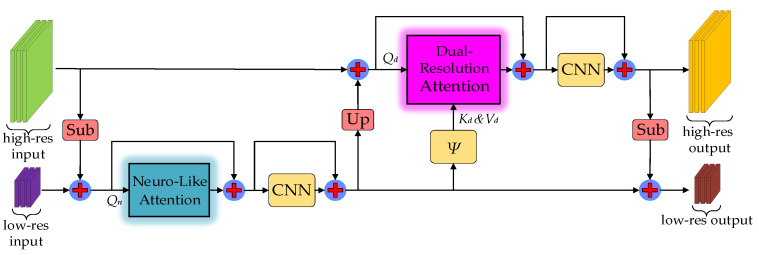
The BatNet block structural diagram.

The difference compared to the external-attention mechanism is that the *K* and *V* parameter matrices in dual-resolution attention are learned from transforming the global context information generated from the low-resolution branch, as shown in Figure 7. It is noteworthy that we only employ the SoftMax function to normalize the attention map, since a SoftMax function performs better than GroupedNorm when the key and value parameter matrices are transformed from the output matrix of the NLA module in the low-resolution branch. Obviously, the multi-head mechanism has been deprecated to reduce system latency.

### 4.2. BatNet Architecture

Before building a complete artificial neural architecture, it is necessary to construct the essential components of the neural network, namely the network block. In order to fuse feature information from different resolution branches, we designed an innovative neural network sub-module with the dual branch structure, dubbed BatNet block, as exhibited in Figure 7. In contrast to the previous works, the BatNet block consists of two types of attention modules along with their convolutional neural network (CNN). Dual-resolution attention and neuro-like attention modules are respectively embedded into high-resolution and low-resolution branches. Neuro-like attention inherits the linear complexity from the EA mechanism, and dispenses with the multi-head mechanism to reduce the system latency on the neuro-like computing architecture. Additionally, the neuro-like attention module reasonably expands the number of learnable parameters in external memory units. We ingeniously arrange the dual-resolution branches into a stepped layout. The *K* and *V* parameter matrices in dual-resolution attention are obtained by transforming the global context information generated from the low-resolution branch. Consequently, the high-resolution branch can capture global contextual information from the low-resolution branch.

In addition to neuro-like attention and dual-resolution attention modules, each of the branches in the BatNet block contains a 3 × 3 convolutional layer without dimension expansion. Conventional transformer-based methods typically use two fully connected layers as feed forward layers, while the feed forward layers expand the input feature dimension by four times. The difference compared to previous transformer-based methods is that the BatNet block has higher execution efficiency than typical transformer-based configurations on parallel computing devices.

**Figure 8:**
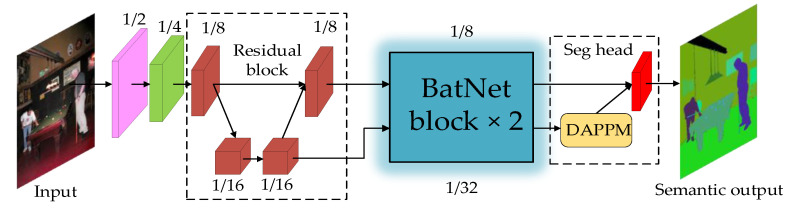
The BatNet architecture illustration.

Based on the BatNet block, we construct a novel Transformer–CNN hybrid neural architecture, named BatNet, for real-time semantic segmentation. Figure 8 illustrates the overall BatNet architecture. At the bottom of the neural network, we project the input image into three channels: red, green, and blue. The first three segments of the network are composed of basic residual modules that consist of a series of 3 × 3 convolutional layers and 1 × 1 convolutional layers. It is noteworthy that, in the third segment, the dual-resolution network structure is applied, and the feature maps are respectively split into high-resolution and low-resolution branches. These can fully integrate local texture information and global semantic information from different resolution branches. For the high-resolution branch, the feature map size is 1/8 of the unchanged input image, while for the low-resolution branch, the feature sizes are 1/16, 1/32, and 1/32, respectively. In order to facilitate the fusion efficiency from different resolution branches, we combine two resolution branches into the stepped layout. The most significant innovation is that the last two segments are constructed of our proposed BatNet block, which enables neural networks to not only extract local geometric textures, but also fuse high-level global contextual information with a smaller number of parameters.

The top of the BatNet is a segmentation head, which is used to predict the category of each pixel. At the end of the low-resolution branch, we inserted a deep aggregation pyramid pooling module (DAPPM) [37,38] to expand the feature map size to match the high-resolution branch. The final feature map size after fusion is 1/8 of the input image. The segmentation head involves a 3 × 3 convolutional layer and a 1 × 1 convolutional layer, while the feature map dimension is the same as that of the input. Ultimately, the output features are categorized at the pixel level to densely predict semantic labels.

We instantiated two different configurations of the network architecture: the original BatNet and the lightweight variant named BatNet-tiny. The original BatNet generates feature maps with more channels than its tiny variant to enrich the feature representation. As recorded in Table 1, we used an array containing five elements to represent the number of feature channels for each segment of the network. Elements containing two numbers represent high-resolution and low-resolution branches, respectively. BatNet-tiny and BatNet have the same network architecture. The difference is that BatNet-tiny only reduces the number of channels by half to further improve the inference speed for real-time semantic segmentation.

## 5. Bat-Inspired Hierarchical 3D Scene Representation

### 5.1. Hierarchical Scene Graph

A scene graph (SG) is a directed acyclic graph commonly used in game engines and 3D modeling. The scene graph is composed of serial vertices and edges where vertices indicate substances in the scene and edges indicate affiliations among vertices. In this section, we propose a layered directed scene graph called a hierarchical scene graph (HSG) enlightened by the mammalian brain mechanism, which is an arborescent and flexible data structure. In general, the root of HSG is at the top of the arborescent graph and the leaves are at the bottom. The hierarchical scene graph decomposes the scene into a hierarchical structure represented by several vertices and edges at different levels of abstraction, where vertices represent the spatial grouping of substances, while edges represent the spatiotemporal relationships between substances, e.g., “There is a black border collie in room A at time t”.

**Figure 9:**
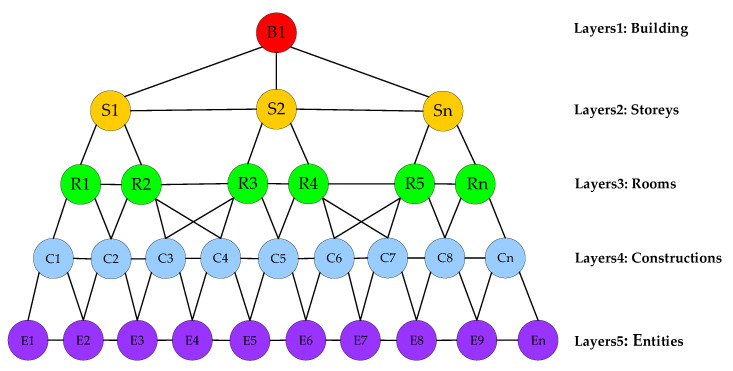
The illustration of hierarchical scene graph. Similar to the analysis of mammalian brains, the hierarchical scene graph decomposes the scene into an arborescent structure represented by serial vertices and edges, where vertices represent the spatial level of substances, while edges represent the spatiotemporal relationships between substances.

The hierarchical scene graph of a multi-story building scene includes five layers from high to low abstraction level: building, stories, rooms, constructions, and entities, as shown in Figure 9. Each abstract or corporeal object in the physical scene has a unique vertex corresponding to it in the hierarchical scene graph. The proposed HSG is constructed with agents’ high-level semantic navigation in mind. For example, consumers can directly issue high-level commands to the agent through dictation: “Take out the kitchen garbage and help me pick up the delivery”. Next, we provide a detailed description of each layer and the vertices they contain.

In the hierarchical scene graph, the upper three layers are abstraction layers and the lower two layers are concrete instances. Since we have assumed that a single building is represented by the HSG, there is only one vertex on the topmost layer, which represents the abstraction concept of the whole building. The building vertex contains the spatial location and semantic labels of the building obtained from the BatNet, and the building edges are connected to all story’s vertices within the story’s layers. Layer 3 in our proposed HSG is the room layer, and the room vertices in this layer are connected to the upper story vertex where the rooms are located. For example, the rooms on the second floor are only connected to the second story vertex, and there is no direct connection to the first and third story vertices. In addition to the rooms, the corridors and stairs are also in layer 3 as they belong to the same abstract level as the room. Layer 4 is the constructions layer, which is composed of wall vertices, floor vertices, ceiling vertices, etc. Moreover, each construction vertex is connected to the nearest room vertex. It is worth noting that the ceiling in a room is the floor of the upper room, so the vertices C3, C4, C6, and C7 in Figure 9 representing the ceiling or floor will be connected to the rooms between two stories. On the contrary, the wall vertices in layer 4 only connect to adjacent rooms. Layer 5 is used to describe specific entities and contains four types of vertices: furniture, agents, pet animals, and persons, whose main distinction is the fact that furniture is stationary, whereas agents, pet animals, and persons are time varying. Edges between different vertices indicate relations, such as relative position, distance, or dependence. For example, edges in layer 5 can represent “there is a gray laptop on the table”, or “the TV on the wall is playing a football game”.

In addition to the arborescent structure, the hierarchical scene graph also has the superiority of flexibility, i.e., the settings for layers, vertices, and edges in the HSG are not stationary and are entirely set according to specific scene-understanding tasks. One can easily insert or discard more layers in the HSG in Figure 9, and can also add or remove vertices or edges. Moreover, we can add further layers at the top, such as the street layer, community layer, and even city layer.

### 5.2. Truncated Signed Distance Field

The truncated signed distance field (TSDF) has recently become a familiar implicit scene volumetric representation for three-dimensional computer reconstruction and game development [39,40,41] since it has several advantages, e.g., uncertainty representation, real-time reconstruction, and ability to generate visible spatial meshes for user monitoring. In contrast to the Euclidean signed distance field (ESDF), the truncated signed distance field leverages the raycasting distance, which is the distance along the viewing ray crossing voxel center to the object surface, and saves this distance information from the Euclidean distance to the transformed truncated distance. Subsequently, the new raycasting points are averaged into the existing TSDF. The strategies for constructing a truncated signed distance field from input pointclouds are extremely significant in terms of both the reconstruction accuracy and the update rate of distance maps. Next, we provide a detailed description of the construction principles for our proposed TSDF.

**Figure 10:**
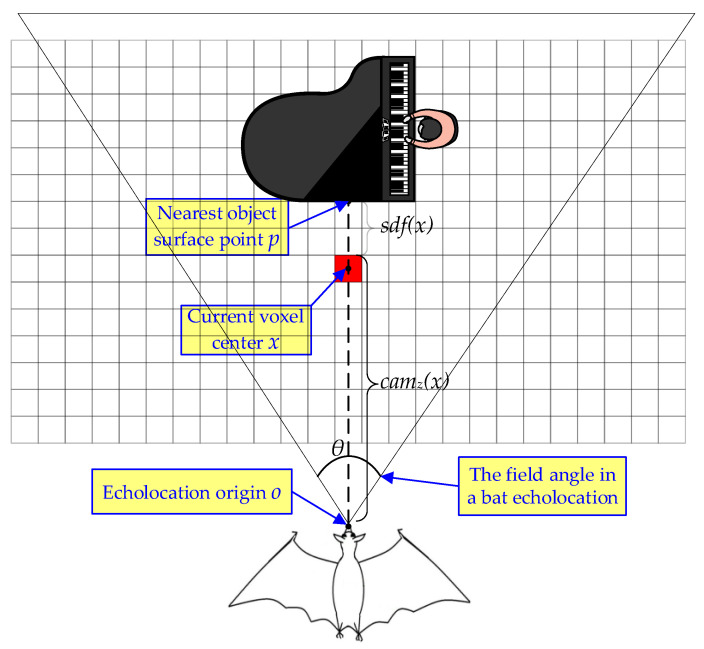
The signed distance field illustration. Each small square represents the spatial voxel in the scene. Like the bat echolocation system, the signed distance field can effortlessly describe obstacle information in the environment and generate a metric distance map, which plays a crucial role in robot autonomous navigation.

Like the bat echolocation system, the signed distance field (SDF) is a set of voxel grids where every voxel element contains its Euclidean distance to the nearest obstacle surface. For an n-dimensional space, the scene is represented through an n-dimensional grid of equally volumetric voxels. Similar to other fields (such as electric field, magnetic field, and gravitational field), the field strength in an SDF is expressed by the Euclidean distance. Each voxel *x* in an SDF contains two types of data, i.e., signed distance sdf*_i_*(*x*) and weight w*_i_*(*x*). The sdf*_i_*(*x*) represents the signed distance information between the voxel center *x* and its nearest object surface along the current raycasting ray, as illustrated in Figure 10. If the spatial position of the voxel center *x* is between the object surface and the sensor origin, the sdf*_i_*(*x*) sign on that side is positive, and vice versa is negative. Given the sensor origin *o*, the position *p* of the nearest pointcloud on the target surface, the current voxel center *x*, and $o,p,x\in {\mathbb{R}}^{3}$, SDF can be formulated as follows:

(9) $${\mathrm{sdf}}_{i}(x)=\left\|p-x\right\|\mathrm{sign}\left(\left(p-x)•(p-o\right)\right)$$

where the subscript *i* denotes the *i*th scan.

For surface reconstruction purposes, the Euclidean distances of sdf*_i_*(*x*) that are too far from the object surface are not instrumental to generating the target mesh. Ultimately, unduly large distances are not conducive to real-time trajectory replanning for robot autonomous navigation. To overcome this disadvantage, the SDF was truncated around the object surface boundary. The truncated variant of sdf*_i_*(*x*) is expressed as follows:

(10) $${\mathrm{tsdf}}_{i}(x)=\max\left(-1,\;\min\left(1,\;\frac{{\mathrm{sdf}}_{i}(x)}{\mathrm{tru}\text{\_}d}\right)\right)$$

where the symbol tru_d represents the truncation distance parameter. In robot navigation applications, the truncation distance tru_d in the TSDF can be understood as the risk coefficient for obstacle distance, which is equal to 1 or −1 to indicate absolute safety. Truncation distance parameter tru_d can be set based on the physical size of the robot.

In TSDF, as mentioned above, there is a weight w*_i_*(*x*) for each voxel to appraise the uncertainty of the corresponding tsdf*_i_*(*x*). Generally, when the updated voxel center *x* is located within the sensor’s field of view, the uncertainty weight w*_i_*(*x*) is set to a constant value of 1. On the contrary, if the voxel center *x* is located outside the sensor’s field of view, the weight w*_i_*(*x*) is set to a constant value of 0.

(11) $$\left\{\begin{array}{l} w_{\mathrm{const}}(x)=1,\mathrm{if}\;x\;\mathrm{within}\;\mathrm{the}\;\mathrm{field}\;\mathrm{of}\;\mathrm{view}\\ w_{\mathrm{const}}(x)=0,\mathrm{else} \end{array}\right.$$

We designed a more sophisticated strategy to combine the uncertainty weights shown above:

(12) $$W_{i}(x)=\min\left(W_{i-1}(x)+w_{i}(x),W_{\max}\right)$$

where W*_i_*(*x*) and w*_i_*(*x*) represent the weights that previously existed in voxels and the weights currently observed, respectively. W_max_ represents the upper limit of all weights, and in our experiment W_max_ = 10,000.

For surface reconstruction requirements, simply finding voxels with truncation distances close to 0 can easily achieve the reconstruction of the entire scene. In order to integrate the TSDF between the previous distance map and current measurements, different observations can be averaged in one TSDF. This is usually done by weighted summation through iterating TSDF as follows:

(13) $${\mathrm{TSDF}}_{i}(x)=\frac{W_{i-1}(x){\mathrm{TSDF}}_{i-1}(x)+w_{i}(x){\mathrm{tsdf}}_{i}(x)}{W_{i}(x)}$$

where TSDF*_i_*(*x*) represents the existing truncated signed distance after *i* iterations for voxel *x* and tsdf*_i_*(*x*) represents current measurements. All voxels are initialized with TSDF*_0_*(*x*) = 0 and W*_0_*(*x*) = 0.

We attempt to facilitate the integration of the new input pointcloud into the existing TSDF by only projecting once per end voxel. We project each input pointcloud into the adjacent voxel and package all pointclouds in the same voxel. We then calculate the average RGB color and distance between the bundled pointclouds, and raycast it only once. Our approach, i.e., packaged raycasting, dramatically promotes raycasting efficiency with only a slight loss in accuracy.

## 6. Experiments

We start by conducting a detailed evaluation of BatNet and its variants, including a detailed model implementation (in Section 6.1.1), am ablation experiment with attention modules (in Section 6.1.2), and analysis with other state-of-the-art approaches (in Section 6.1.3), in order to demonstrate the superiority of our proposed bat-inspired hybrid architecture. Subsequently, we conducted more comprehensive experiments for scene understanding, including TSDF-based metric scene reconstruction (in Section 6.2.1), bat-inspired volumetric-semantic scene understanding (in Section 6.2.2), and masking for a time-varying target (in Section 6.2.3). Finally, we utilized our self-developed drone to perform a 3D geometric–semantic scene-understanding test in the real world (in Section 6.3). The evaluation for robot state estimation has already been performed in our previous work [32,33,34,35], so is not a contribution of this article.

### 6.1. Image Segmentation

#### 6.1.1. Datasets and Implementation Details

The Cambridge-driving Labeled Video Database (CamVid) is a road scene segmentation dataset collected from an automobile camera. It contains 701 images with high-quality dense pixel-level annotations. These images with a resolution of 960 × 720 are respectively split into 367 for semantic segmentation model training, 101 for validating, and 233 for testing. The annotated images provide 32 candidate classes, of which the subset of just 11 categories is used in our experiments for fair comparison with other neural architectures. In this article, we combine the 367 training images and 101 validating images for training BatNet, and the 233 testing images are used to evaluate BatNet.

Cityscapes [42] is a large-scale dataset that focuses on scene understanding in an urban street background. The dataset contains 5000 annotated high-resolution images, which are further split into 2975, 500, and 1525 images for neural network training, validating, and testing respectively. Incredibly, the image resolution in the Cityscapes dataset has reached 2048 × 1024, which is exceptionally challenging for real-time scene-understanding scenarios. The annotated images have 30 different categories, but just 19 categories are used in our experiments for a fair comparison with other image segmentation methods.

ADE20K is an enormous dataset used for scene understanding and contains 25 K images and 150 fine-grained semantic categories. All images in ADE20K are fully annotated with objects, and are split so that with 20 K used for semantic segmentation model training, 2 K used for validating, and 3 K used for testing. Due to numerous categories and challenging scenes, this dataset is quite challenging for real-time semantic segmentation methods.

In this section, we conduct all experiments based on PyTorch 1.8. The performance compared with other scene-understanding approaches was evaluated by running on a single NVIDIA GeForce 1660 GPU with CUDA 10.2, CUDNN 7.6. The artificial neural networks were trained from scratch with the initial learning rate of 0.001 and the weight decay of 0.05. We trained all neural networks with the AdamW optimizer and adopted the poly learning rate scheduler with the power of 0.9 to drop the learning rate. For data augmentation, we conducted random scaling, random cropping, random color jittering, and random horizontal flipping. The cropped resolution for the Cityscapes dataset was 1024 × 512, for the CamVid dataset was 960 × 720, and for the ADE20K dataset was 512 × 512. The random scale ranges were within [0.5, 0.75, 1.0, 1.25, 1.5]. We applied the standard mean intersection over union (mIoU) for segmentation accuracy comparison and frames per second (FPS) for inference speed comparison.

#### 6.1.2. Ablation Study

Ablation studies were conducted to demonstrate the performance of our proposed modules and to dissect these improvements. The ablation experiment selects BatNet-tiny as the basic model and uses the same neural network training setting on the ADE20K dataset. Table 2 displays the quantitative model performance and operational efficiency with ablating modules.

Conventional transformer-based methods typically use two fully connected layers as feed forward layers, but our proposed network block uses convolutional neural layers. The ablation studies show that our proposed convolutional layers outperform typical feed forward layers, not only for segmentation accuracy, but also for inferential efficiency. To validate the superiority of our two proposed attention mechanisms, we implement the different attention mechanisms under identical experimental conditions. We find that neuro-like attention outperforms other forms of multi-head-based external attention, and is much more efficient than the traditional self-attention mechanism. When we replaced the attention mechanism with dual-resolution attention in the high-resolution branch, the accuracy improved further, with reasonable latency. The ablation experiment implies that our proposed two attention mechanisms achieve a better trade-off between segmentation accuracy and inferential efficiency than multi-head-based attention mechanisms on neural computing platforms.

#### 6.1.3. Comparison with State-of-the-Art Approaches

We compare the segmentation accuracy and inference speed of our proposed BatNet with previous state-of-the-art real-time neural networks on the CamVid test set. A detailed description is exhibited in Table 3. Model performances are evaluated with a single crop of 960 × 720, and FPS is estimated under the same input scale. On CamVid with the input size of 960 × 720, BatNet achieved the highest image segmentation accuracy, while its lightweight variant, BatNet-tiny, achieved the fastest inference speed. The experimental results demonstrate that the bionics-based BatNet architecture achieves a state-of-the-art trade-off between performance and inference efficiency compared to other methods.

To further evaluate the real-time performance of BatNet, we also conducted experiments on the high-resolution Cityscapes dataset. It can be seen from Table 4 that BatNet achieved 76.1% mIoU, far surpassing other semantic segmentation models. At the same time, the lightweight variant of BatNet, BatNet-tiny, achieved 49.6 FPS with only 4.9 M parameters. These experimental results demonstrate that the BatNet architecture maintains an excellent balance among accuracy, model capacity, and operational efficiency, even when applied to high-resolution images.

To validate the generalization ability of scene-understanding models on large-scale datasets, we conducted comparative experiments with other state-of-the-art models on ADE20K. Due to the large number of images and excessive categories, ADE20K is almost unfeasible for lightweight scene-understanding models. Table 5 presents the comparison of BatNet with state-of-the-art scene-understanding models, including both efficient convolutional neural networks and lightweight vision transformer-based models, and reports the results for accuracy, model size, and inference speed. As the results show, BatNet achieves superior comprehensive characteristics on large-scale datasets, and outperforms other state-of-the-art methods, not only for mIoU, but also for model size, while maintaining a competitive edge in FPS.

### 6.2. Scene Representation

#### 6.2.1. TSDF-Based Volumetric Scene Reconstruction

**Figure 11:**
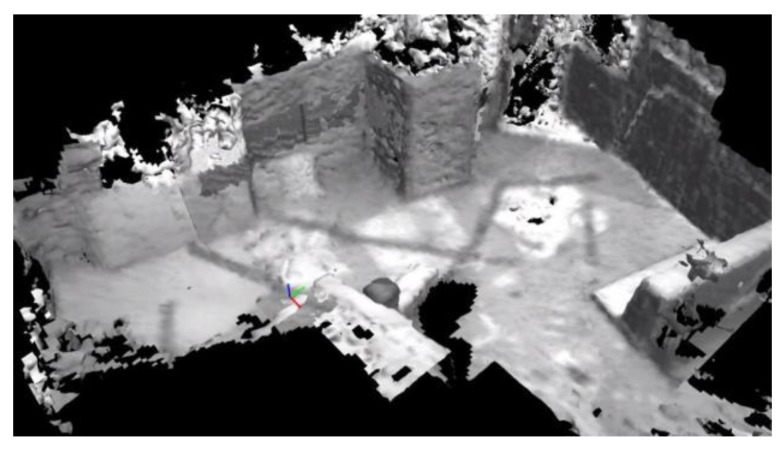
The qualitative reconstruction result with TSDF-based surface mesh production from the EuRoC_v1 sequence.

Surface mesh production is a common means of scene description, and realizes scene representation by iterative reconstruction of entities in the environment. Next, we qualitatively demonstrate the performance of our proposed TSDF-based method by reconstructing the V1 sequence in the EuRoC database. The EuRoC datasets [43] are collected from a stereo camera and a synchronized inertial measurement unit carried by an agile unmanned aerial vehicle (UAV). We use the stereo image matching toolbox contained in the robot operating system (ROS) to convert binocular vision into spatial pointclouds. We employ the self-developed LDMF state estimation system [35] as the odometer for UAV pose estimation. All operations in this section were executed using an NVIDIA Jetson Xavier NX Embedded computer. The voxel size in TSDF was set to 20 cm, truncation distance was set to 1 meter, max ray length was equal to 6 meters, and maximum weight W_max_ = 10,000 for Formula (12). The qualitative reconstruction is shown in Figure 11.

#### 6.2.2. Bat-Inspired Volumetric–Semantic Scene Representation

After verifying the reconstruction effect in the previous section, we utilize BatNet-tiny to achieve pixel-level image semantic segmentation, and semantically propagate a label to each spatial mesh. Subsequently, each spatial voxel has a vector of semantic category probabilities. We validated the environmental perception effect with BatNet-tiny and TSDF using two publicly available datasets, uHumans2 and KITTI.

**Figure 12:**
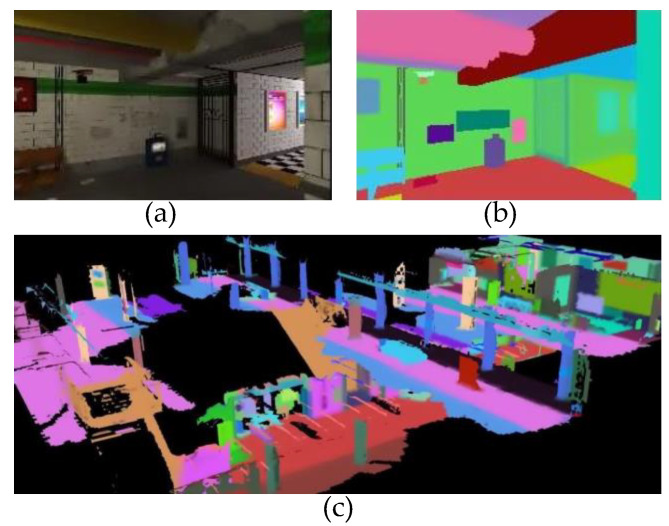
The qualitative reconstruction generated by our proposed volumetric–semantic scene representation system. (**a**) The colorful image stream is manufactured by the “subway” sequence. (**b**) The semantic image corresponds to (**a**), and the different colors in the semantic image represent the corresponding categories. (**c**) Volumetric–semantic scene reconstruction.

uHumans2 is a virtual dataset created by a computer simulator, which is collected by a photo-realistic Unity-based game engine provided by MIT Lincoln Laboratory. The uHumans2 dataset provides RGB images, depth images, IMU, scan, and semantic annotations of the scenario. In the uHumans2 dataset, we thoughtfully selected the “subway” sequence, a super large-scale multi-floor scene, as the subject of environmental perception experiments, which is very challenging for lightweight scene-understanding systems. Since the dataset already contains semantically segmented images, we do not need to use BatNet-tiny for image semantic segmentation. The voxel size in TSDF is set to 15 cm, the truncation distance is set to 2, the max ray length is equal to 10 meters, and the maximum weight W_max_ = 10,000 for Formula (12). All operations were executed using an NVIDIA Jetson Xavier NX Embedded computer. The qualitative reconstruction is shown in Figure 12.

**Figure 13:**
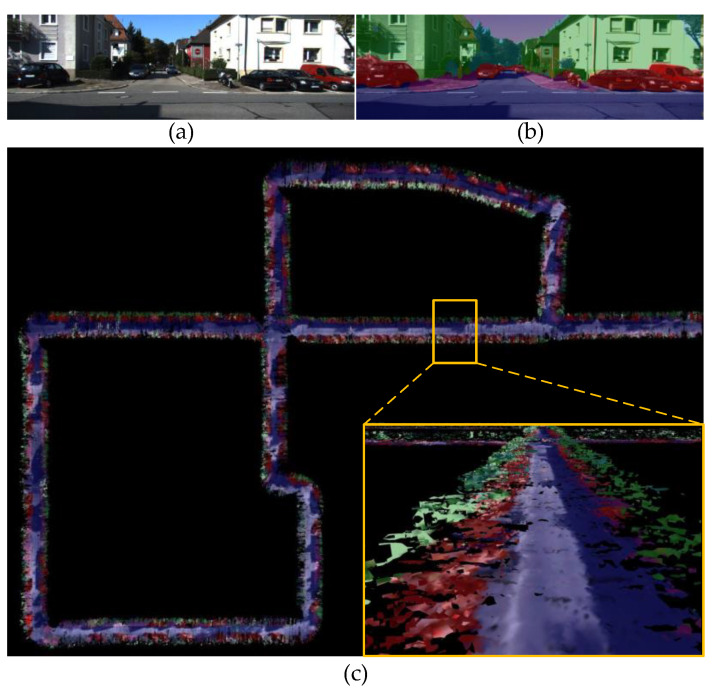
The top view of a reconstructed community street in the KITTI dataset. (**a**) The RGB image stream. (**b**) The semantic image produced by our proposed BatNet-tiny. (**c**) Volumetric–semantic scene reconstruction.

KITTI is a challenging real-world computer vision dataset used for 3D object detection, visual–inertial odometry, and stereo tracking, and is collected by two high-resolution binocular cameras, a high-precision inertial measurement unit, a Velodyne laser, and a RTK localization system. KITTI is captured by driving around a medium-scale urban environment, on community streets and on highways. Limited by the computational power of our NVIDIA Jetson Xavier NX chip, we used BatNet-tiny to asynchronously segment images on a desktop computer with an NVIDIA GeForce 1660 GPU to obtain pixel-level semantic annotations. Then, the obtained semantic image was aligned with the timestamp of the original RGB image. The configuration related to TSDF reconstruction is identical to that of the uHumans2 dataset. The top view of a reconstructed community street in KITTI is shown in Figure 13.

#### 6.2.3. Visualization of Hierarchical Scene Graph

**Figure 14:**
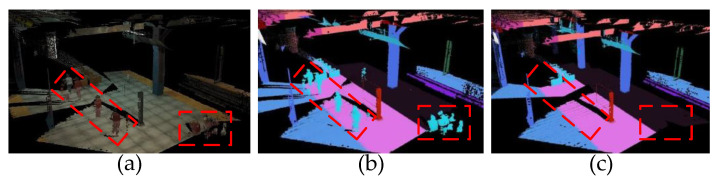
The qualitative comparison of different results when discarding a vertex from layer 5 in the hierarchical scene graph. (**a**) The geometric scene reconstruction. (**b**) The geometric–semantic scene reconstruction. (**c**) The geometric–semantic scene reconstruction with HSG which removed “person” vertices from layer 5.

**Figure 15:**
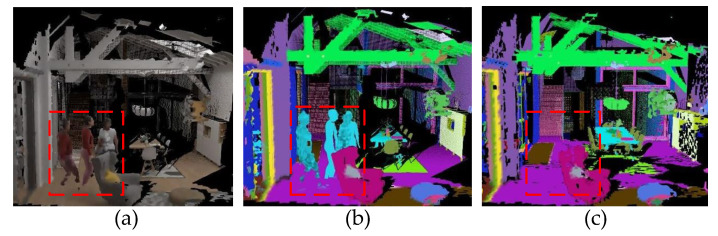
The qualitative comparison of masking for time-varying targets. (**a**) The geometric scene reconstruction. (**b**) The geometric–semantic scene reconstruction. (**c**) The geometric–semantic scene reconstruction with HSG which removed “person” vertices from layer 5. The pedestrians are moving from left to right when reconstructing this scene.

In this section, we visualize the effect of time-varying vertices from layer 5 in the hierarchical scene graph. As we mentioned in Section 5.1, we include three types of time-varying vertices, i.e., agents, pet animals, and persons. Without loss of generality, here we use pedestrians as a typical case to demonstrate the approach’s effectiveness. In order to eliminate interference from other external factors, we choose the virtual uHumans2 created by computer simulator for the experiment. As shown in Figure 14 and Figure 15, when the “person” vertices are discarded from layer 5 in the hierarchical scene graph, the corresponding category of meshes in the scene also disappears.

### 6.3. Real-World Experiment

**Figure 16:**
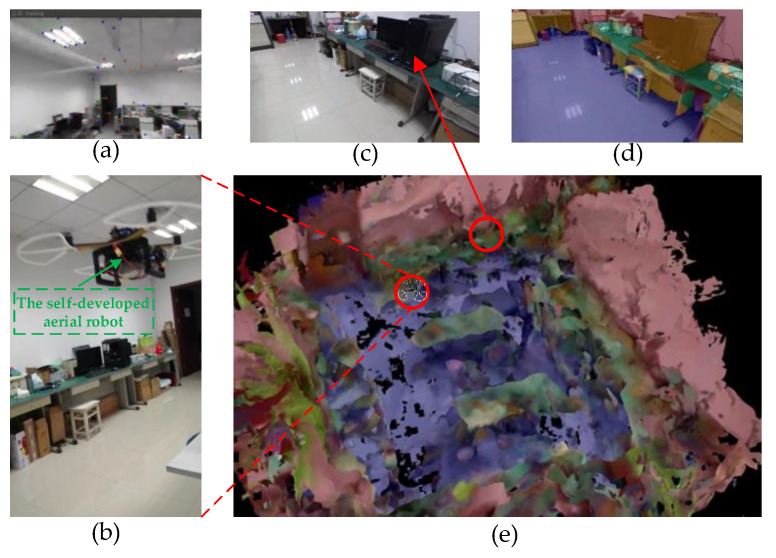
The real-world geometric–semantic scene representation experiments. (**a**) The visual feature state is transmitted from the self-developed drone. (**b**) The drone is collecting scene information in our office. (**c**) The RGB image frame was collected by the airborne ZED camera. (**d**) The semantic image produced by the proposed BatNet-tiny. (**e**) The volumetric–semantic scene reconstruction.

In order to verify the practicability of our proposed scene-understanding method, we also conducted real-world semantic scene reconstruction in our office with a self-developed drone, as shown in Figure 16. We directly leveraged the stereo image matching kit to convert RGB and depth images collected by the ZED camera into spatial pointclouds. We employed the self-developed LDMF state estimation system [35] as the visual–inertial odometry for drone pose estimation. We utilized BatNet-tiny to achieve pixel-level image semantic segmentation, and semantically propagated a label to each spatial mesh. Subsequently, each spatial voxel has a vector of semantic category probabilities. The voxel size in TSDF was set to 30 cm, truncation distance was set to 1, max ray length was equal to 5 m, and maximum weight W_max_ = 10,000.

## 7. Conclusions and Future Work

This article proposed a novel scene-understanding system that seamlessly captures metric and semantic aspects of an unexplored environment. Our evaluation shows that the proposed scene-understanding system achieves competitive performance in image semantic segmentation and volumetric–semantic scene reconstruction. Moreover, to verify the practicability of our proposed scene-understanding method, we also conducted real-world semantic scene reconstruction in an indoor environment with our self-developed drone.

Although the algorithm proposed in this article was verified to a certain extent in physical experiments, there is still a significant gap between it and consumer-grade robot applications. With the gradual improvement in ethical constraints and legal regulations related to robots in the future, the application of intelligent robots and autonomous vehicles will become increasingly widespread.

## Figures and Tables

**Table 1:** The different configurations of BatNet architecture.

Networks	Channel Number	Parameters
BatNet	[64, 128, 128/256, 128/512, 128/512]	17.1 M
BatNet-tiny	[32, 64, 64/128, 64/256, 64/256]	4.9 M

**Table 2:** Ablation studies for our proposed modules on the ADE20K dataset. The capital letters SA, EA, NLA, and DRA represent self-attention, external attention, neuro-like attention, and dual-resolution attention, respectively.

Convolutional FFN	SA	EA	NLA	DRA	mIoU (%)	FPS
✓	✓	✗	✗	✗	32.6	71.3
✓	✗	✓	✗	✗	31.4	131.5
✓	✗	✗	✓	✗	32.5	142.7
✗	✗	✗	✓	✓	32.3	130.4
✓	✗	✗	✓	✓	32.8	138.1

**Table 3:** Comparison with state-of-the-art approaches on the CamVid test set.

Model	Backbone	mIoU (%)	Parameters	FPS
BiSeNet	Xception	62.4	5.8 M	75.3
BiSeNetV2	Booster	69.3	-	68.1
SFNet	ResNet18	70.9	12.9 M	46.3
STDCSeg	STDC1	68.8	14.2 M	85.3
STDCSeg	STDC2	70.7	22.2 M	63.6
BatNet-tiny	HybridBlock	77.9	4.9 M	114.4
BatNet	HybridBlock	80.2	17.1 M	68.3

**Table 4:** Comparison with state-of-the-art approaches on the Cityscapes validation set. The model performances are estimated with a single crop of a 2048 × 1024 resolution.

Model	Backbone	mIoU (%)	Parameters	FPS
BiSeNet	Xception	66.1	5.8 M	49.4
BiSeNetV2	Booster	70.5	-	45.2
SFNet	ResNet18	72.9	12.9 M	8.2
STDCSeg	STDC1	71.7	14.2 M	38.3
STDCSeg	STDC2	73.2	22.2 M	33.5
BatNet-tiny	HybridBlock	73.3	4.9 M	49.6
BatNet	HybridBlock	76.1	17.1 M	27.2

**Table 5:** Comparison with state-of-the-art approaches on the ADE20K validation set.

Model	Backbone	mIoU (%)	Parameters	FPS
FCN	MobileNetV2	18.1	9.8 M	47.4
DeepLabV3	MobileNetV2	30.5	15.4 M	32.9
BiSeNetV2	Booster	31.4	-	95.3
SegFormer	MiT-B0	35.7	3.8 M	42.8
BatNet-tiny	HybridBlock	32.8	4.9 M	138.1
BatNet	HybridBlock	38.5	17.1 M	69.2

## Data Availability

The CamVid dataset: http://mi.eng.cam.ac.uk/research/projects/VideoRec/CamVid/ (accessed on 21 June 2023); the Cityscapes dataset: https://www.cityscapes-dataset.com (accessed on 21 June 2023); the ADE20K dataset: http://groups.csail.mit.edu/vision/datasets/ADE20K/ (accessed on 20 June 2023); the EUROC dataset: https://projects.asl.ethz.ch/datasets/doku.php?id=kmavvisualinertialdatasets (21 December 2021); the KITTI dataset: https://www.cvlibs.net/datasets/kitti/raw_data.php (accessed on 20 June 2023).
